# Study of p-SiC/n-GaN Hetero-Structural Double-Drift Region IMPATT Diode

**DOI:** 10.3390/mi12080919

**Published:** 2021-07-31

**Authors:** Yang Dai, Qingsong Ye, Jiangtao Dang, Zhaoyang Lu, Weiwei Zhang, Xiaoyi Lei, Yunyao Zhang, Han Zhang, Chenguang Liao, Yang Li, Wu Zhao

**Affiliations:** 1 School of Information Science and Technology, Northwest University, Xi’an 710127, China; yeqingsong971020@foxmail.com (Q.Y.); dang101713@foxmail.com (J.D.); luzy7@foxmail.com (Z.L.); leixy@nwu.edu.cn (X.L.); yunyaozhang@nwu.edu.cn (Y.Z.); hanzhang@nwu.edu.cn (H.Z.); nicoker@nwu.edu.cn (C.L.); zhaowu@nwu.edu.cn (W.Z.); 2 Shanghai Precision Metrology and Testing Research Institute, Shanghai 201109, China; zhangww0702@sina.com; 3 School of Microelectronics, Xidian University, Xi’an 710071, China

**Keywords:** double-drift region (DDR), Heterostructure, silicon carbide (SiC), gallium nitride (GaN), impact ionization avalanche transit time (IMPATT)

## Abstract

Nowadays, the immature p-GaN processes cannot meet the manufacturing requirements of GaN impact ionization avalanche transit time (IMPATT) diodes. Against this backdrop, the performance of wide-bandgap p-SiC/n-GaN heterojunction double-drift region (DDR) IMPATT diode is investigated in this paper for the first time. The direct-current (DC) steady-state, small-signal and large-signal characteristics are numerically simulated. The results show that compared with the conventional GaN single-drift region (SDR) IMPATT diode, the performance of the p-SiC/n-GaN DDR IMPATT proposed in this design, such as breakdown voltage, negative conductance, voltage modulation factor, radio frequency (RF) power and DC-RF conversion efficiency have been significantly improved. At the same time, the structure proposed in this design has a larger frequency bandwidth. Due to its greater potential in the RF power density, which is 1.97 MW/cm^2^ in this study, indicates that the p-SiC/n-GaN heterojunction provides new possibilities for the design and manufacture of IMPATT diode.

## 1. Introduction

As one of the main microwave and terahertz wave solid-state sources, impact ionization avalanche transit time (IMPATT) diodes are expected to have broad development prospects in this field due to their better RF performance [1,2]. Material properties and structural design will seriously impact on the performance of IMPATT diode. Based on the fact that silicon carbide (SiC) and gallium nitride (GaN) materials have higher breakdown voltage and faster electron velocity, they are applicable for manufacturing the IMPATT device [3,4,5,6,7]. Conventional GaN-based SDR IMPATT diodes are mainly composed of the avalanche p-n junction and carrier drift region, but the double-drift region (DDR) diode consists of a p-n junction and two drift regions, and both holes and electrons can be drifted to generate a better RF performance [8,9,10,11]. In terms of conventional SDR IMPATT diode, the higher the hole concentration of p-type GaN, the better the RF performance of the diode [12,13]. Therefore, before the performance of the IMPATT diode is saturated, as the p-type doping concentration increases, the performance of the device is enhanced accordingly.

However, owing to the difficulty in the doping manufacturing process of p-GaN, a high-concentration of ionized holes cannot be formed [14], leading to a series of problems. Firstly, because of the overly low p-type region concentration and poor mobility characteristics, the electric field distribution in the avalanche region deviates from the ideal situation, deteriorating the impact-ionzation characteristics and inducing the device performance to decline. Secondly, the p-GaN series resistance is too high, which undermines the device performance and stops it working in some cases [15]. It is precisely because of these problems that the DDR IMPATT diode based on the GaN material has not been realized. In previous simulation studies on GaN-material DDR IMPATT diodes, the ionized hole concentration is usually set to a higher ideal value [16,17], and it often reaches the order of 10^19^ cm^−3^ or even 10^20^ cm^−3^. As GaN-based DDR IMPATT diodes are impossible to achieve in the manufacturing process, the current research of GaN-based IMPATT device is mainly based on single-drift structures.

The manufacturing process of p-SiC is relatively mature [18], with an ionized hole concentration reaching 1 × 10^19^ cm^−3^, and it can meet the working requirements of IMPATT devices. That is why this paper proposes a new type of p-SiC/n-GaN DDR IMPATT diode, which opts for p-SiC instead of p-GaN to construct the p-type avalanche and drift regions. Since there is not much difference between the bandgap widths of SiC and that of GaN material, each close to 3.4 eV, the heterojunction interface formed by SiC and GaN has no significant effect on the discontinuity of the energy band. Despite that, there is certain interface mismatch between SiC and GaN, the technology for growing GaN on SiC substrates is relatively mature, which can minimize the influence on the device [19,20,21]. Since this is the first study of the performance of this structure in IMPATT device, the interface mismatch at the heterojunction is ignored and the performance of the new structure is explored under ideal conditions in this study. As the results revealed, compared with the conventional SDR IMPATT diode, the RF power of the p-SiC/n-GaN DDR IMPATT device is significantly increased due to the hole participation in the avalanche-transition process.

## 2. Simulation Models and Methods

A variety of models including the drift-diffusion model, recombination model, impact-ionization model, GaN and SiC mobility models are applied in the simulation to theoretically characterize the DC and RF performance of the p-SiC/n-GaN DDR IMPATT. The device is characterized by constructing the physical structure, material parameters and physical model of the diode. In this study, the performance of the conventional GaN-based SDR IMPATT diode and the new structure SiC/GaN heterojunction DDR IMPATT diode was studied simultaneously to facilitate the comparison.

**Figure 1:**
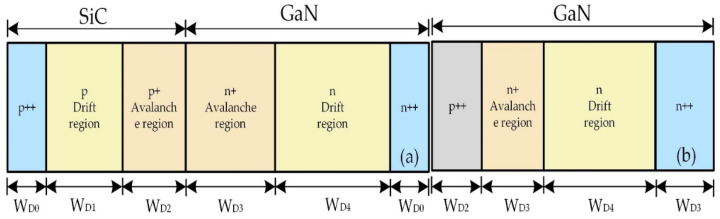
(**a**) The DDR IMPATT diode and (**b**) SDR IMPATT diode structure comparison diagram.

As shown in Figure 1, it is a structural diagram of DDR IMPATT diode and SDR IMPATT diode. Table 1 displays the structural parameters of the doping concentration and thickness of each layer of IMPATT diodes with different structures. As the hole drift velocity of 6H-SiC is faster than that of 4H-SiC, 6H-SiC and wurtzite GaN are selected in this study to design the heterojunction IMPATT. The DDR IMPATT structure is composed of SiC and GaN materials, and the SDR IMPATT is composed of all GaN materials. The cross-sectional area of the diode is 100 μm^2^, the DC bias current density J_dc_ of the device is 100 kA/cm^2^, and the simulated temperature is 300 K.

**Figure 2:**
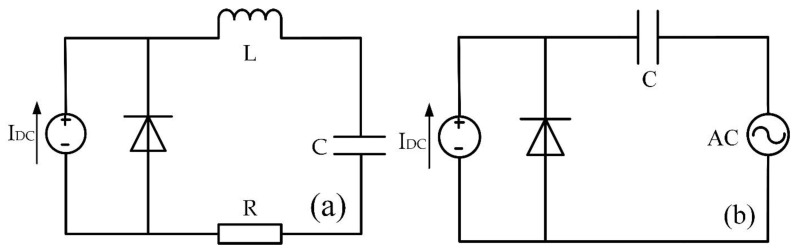
(**a**) The free-running and (**b**) voltage-driven oscillation circuit diagram.

As shown in Figure 2, the large-signal RF oscillation circuit of the device adopts two kinds of free-running oscillation circuit and voltage-driven oscillation circuit. I_DC_ is a DC current source with continuous output. The simulation is based on the Silvaco simulation software. The Silvaco mixed-mode module and Silvaco atlas module are chosen for “device-circuit” mixed simulation and device simulation respectively. Impact-ionization and mobility models were derived from references [22,23,24], where a and b signifies impact-ionization coefficients and *E* symbolizes the electric field intensity.

(1) $$\alpha_{n,p}=a_{n,p}exp\left(-\frac{b_{n,p}}{E}\right)$$

Considering the existence of SiC/GaN heterojunction, there is a tunneling current from band to band under high field. The tunneling current model comes from references [25,26]. In this study, in the presence of a certain tunneling current, the performance of the device is further studied.

(2) $$J_{te}=AE^{\lambda }exp\left(-\frac{B}{E}\right)$$

The GaN negative differential mobility model is derived from references [27,28]. Unlike the constant mobility models of other materials, GaN materials have obvious negative differential mobility characteristics, which is also beneficial to the performance of the device [29]. The relevant parameters involved in the above model are showcased in Table 2.

(3) $$\mu \left(E\right)=\frac{\mu_{0}\left(N\right)+v_{sat}\frac{E^{\delta -1}}{E_{c}{}^{\delta }}}{1+\alpha {\left(\frac{E}{E_{c}}\right)}^{\gamma }+{\left(\frac{E}{E_{c}}\right)}^{\delta }}$$

In the free-running oscillation circuit in Figure 2a, the operating frequency band of the IMPATT diode can be obtained by repeatedly adjusting the RLC parameters of the circuit through trial and error. When the free-running oscillation circuit is able to oscillate steadily, the maximum RF performance of IMPATT device can be obtained by adjusting the load resistance. In the voltage-driven oscillation circuit in Figure 2b, the operating frequency and the amplitude of the RF voltage of the device can be controlled by the RF voltage source. Therefore, the voltage modulation coefficient *m* = V_RF_/V_DC_ can be adjusted by controlling the amplitude of the RF voltage source directly. Next, the Fourier analysis on the stable oscillation waveform data is conducted through Matlab to gain the main RF performance indicators such as the frequency, RF power and efficiency of the diode.

## 3. Simulation of the DDR IMPATT Device and Discussion

### 3.1. DC Simulation Results and Analysis

**Figure 3:**
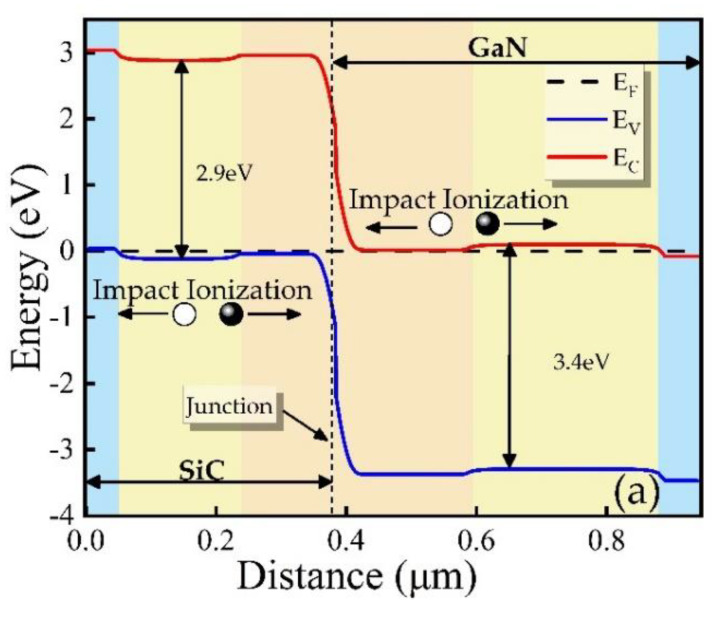

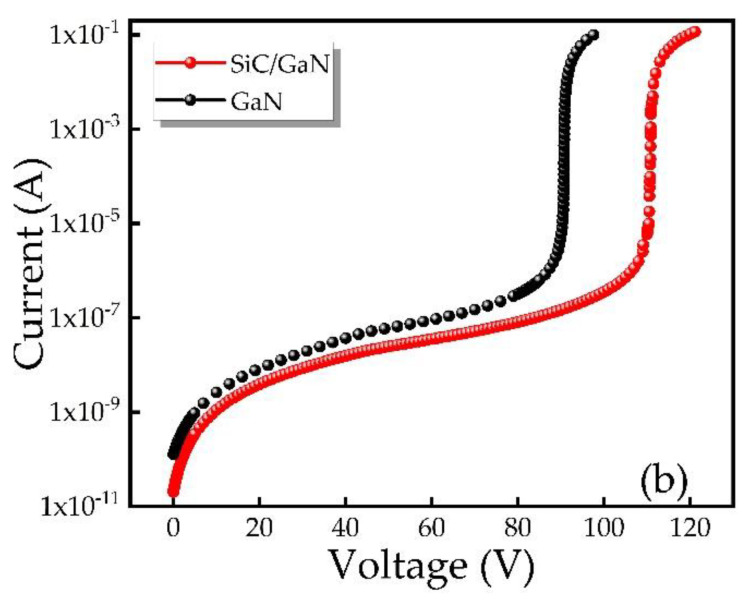
(**a**) DDR IMPATT energy band diagram of the thermal equilibrium state and (**b**) breakdown characteristic of different IMPATT diodes.

Figure 3a is the energy band distribution diagram of p-SiC/n-GaN DDR IMPATT diode. Avalanche ionization will occur at the DDR heterojunction interface to produce electron-hole pairs. When an avalanche occurs, the injected electrons and holes drifted by the action of an electric field, electrons flow from SiC to GaN, and holes flow from GaN to SiC, thereby generating RF current. In Figure 3b, the breakdown characteristics of the devices are plotted on a logarithmic scale. The IMPATT is deemed to be “breakdown” when the reverse saturation current reaches 10^−5^ A as defined in this work, and the cathode voltage at this current is the breakdown voltage. Through the experiment, it can be seen that the breakdown voltage of the GaN-based SDR IMPATT is 90.8 V, while the breakdown voltage of the designed DDR IMPATT is higher and can reach 110 V.

**Figure 4:**
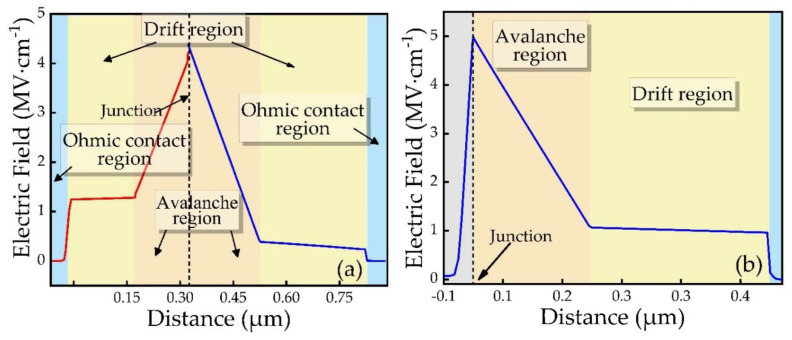
Electric field of (**a**) DDR IMPATT and (**b**) SDR IMPATT as a function of distance.

Figure 4 is the distribution diagram of the internal electric field distribution of different diodes when the reverse current reaches 100 kA/cm^2^. It can be observed that the electric field distribution has reached the design expected effect. Part of the electric field falls in both the p-type region and the n-type region. The electric field intensity in the avalanche region is higher. Since the avalanche region is uniformly doped with high concentration, and the electric field distribution is a triangular field. For SDR IMPATT diodes, the electric field almost all falls on the n-type side. The SDR IMPATT avalanche region is a triangular field, and its peak electric field is greater than that of the DDR IMPATT avalanche region, which means that the SDR IMPATT is more prone to breakdown.

**Figure 5:**
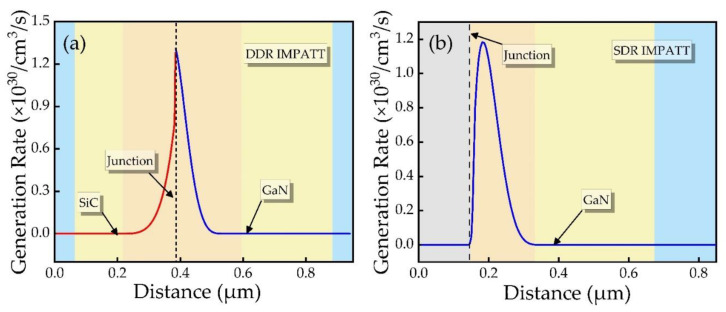
Impact-ionization generation rate as a function of distance. (**a**) SiC/GaN DDR, (**b**) GaN SDR.

Figure 5 shows the impact-ionization rate schematic diagram of SiC/GaN DDR and GaN SDR IMPATT under breakdown conditions. It can be seen from the Figure 5a that the impact-ionization rate curve meets the design expectations, and the avalanche is well confined in the avalanche region. At the heterojunction interface, the peak impact-ionization rate on the GaN side is 1.31 × 10^30^ cm^−3^s^−1^, and the peak value on the SiC side is 1.28 × 10^30^ cm^−3^s^−1^. As can be seen from the result of DC characteristic analysis, it is feasible to adopt the DDR heterojunction instead of SDR junction while designing the IMPATT device, and it can also make the device work in IMPATT mode.

### 3.2. Small-Signal Simulation Results and Analysis

**Figure 6:**
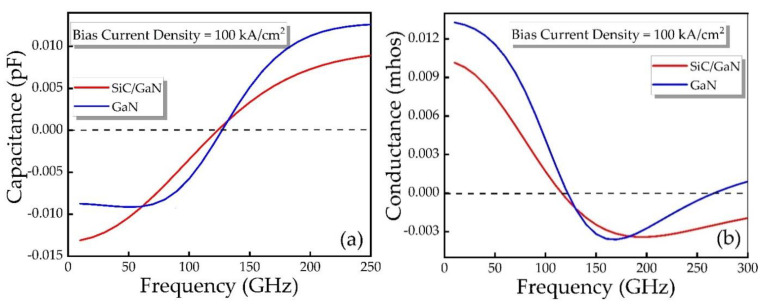
(**a**) Capacitance and (**b**) conductance as a function of frequency for different IMPATT diodes.

Figure 6 reveals the capacitance and conductance as a function frequency obtained through small-signal simulation for different IMPATT devices. It can be seen from the Figure that the DDR IMPATT and the conventional SDR IMPATT have almost the same change trends in capacitance and conductance. As the frequency increases, the capacitance continues to rise. It is found that SDR IMPATT diode has a negative conductivity band of 120 to 250 GHz, while DDR IMPATT diode has a negative conductivity band of 120 to 300 GHz. Meanwhile, compared with SDR IMPATT diode, DDR IMPATT diode has a larger-signal working frequency band.

**Figure 7:**
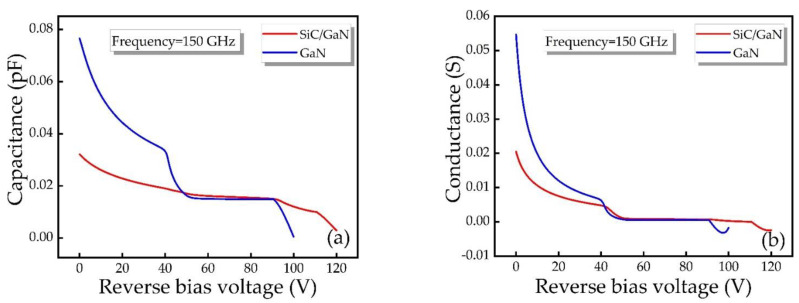
(**a**) Capacitance and (**b**) conductance as a function of bias voltage for different IMPATT diodes at 150 GHz.

Figure 7 shows the capacitance and conductance as a function of the bias voltage for different diodes at 150 GHz. As the voltage increases, the capacitance and conductance gradually decrease. As can be seen from the Figure, when the voltage is greater than the breakdown voltage, the conductance is negative, indicating that the DDR IMPATT diode, as a negative-resistance device, can only operate under voltage after the breakdown.

### 3.3. Large-Signal Simulation Results and Analysis

**Figure 8:**
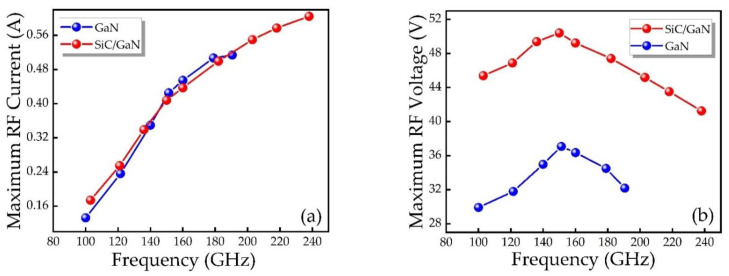
(**a**) RF current and (**b**) RF voltage as a function of frequency of different IMPATT diodes.

As a first step, a free-running circuit is utilized to study the RF output characteristics of IMPATT diodes with different structures. Figure 8 explains the relationship between the maximum RF current amplitude, RF voltage amplitude and frequency of IMPATT diodes with different structures. The DC bias current density is set to 100 kA/cm^2^. As shown in Figure 8a, the RF current amplitudes of IMPATT diodes of different structures demonstrate the same changing trend with frequency, and the alternating current amplitude increases with increasing frequency. There is no significant difference in the RF current amplitude of the two structures. The reason is that whether it is a DDR or SDR IMPATT devices, the total current is the sum of the hole current and the electron current generated by the avalanche. However, in SDR structure, the hole current is directly absorbed by the electrode. According to Figure 8b, as the frequency increases, the RF voltage amplitude first increases, and then begins to decrease when the frequency is greater than 150 GHz. At the same frequency, the RF voltage amplitude of the DDR IMPATT device is significantly greater than that of the SDR IMPATT device. The amplitude of SDR is 37 V at 150 GHz, but DDR can reach 50 V, indicating that the RF voltage of DDR IMPATT is significantly higher than that of SDR IMPATT. Based on the above analysis, it can be speculated that the RF power of DDR IMPATT should be better than that of SDR IMPATT.

**Figure 9:**
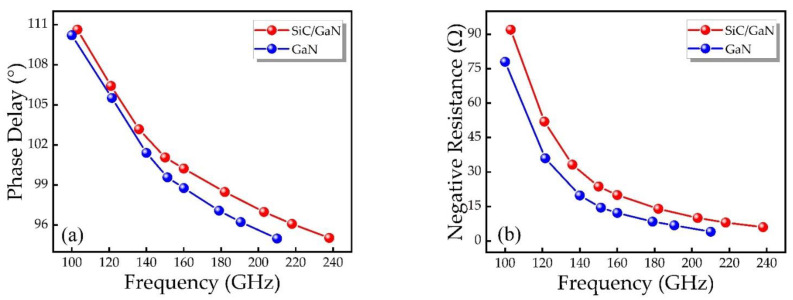
The relationship between (**a**) phase delay, (**b**) negative resistance and frequency of different IMPATT diodes.

Figure 9a displays the relationship between the RF voltage-current phase delay and the RF operating frequency of the two structures. The phase delay will lead to differences in the RF performance of IMPATT devices with different structures. It can be seen from Figure 9a that the phase delay decreases with the rise of frequency. DDR IMPATT diode is slightly higher than SDR IMPATT diode. According to the RF power formula [30]:

(4) $$P_{RF}=\frac{1}{2}\cdot V_{RF}\cdot I_{RF}\cdot \left|\cos\theta \right|$$

The phase delay *θ* in the new structure is more beneficial to device performance. For IMPATT devices, when the phase delay is less than 90°, the device will not work properly. It can be seen from Figure 9a that the failure operating frequency point of DDR IMPATT is higher than that of SDR IMPATT. When the frequencies of SDR structure and DDR structure are respectively 200 GHz and 240 GHz, the phase delays decay to 95°. Obviously, DDR IMPATT diode have more advantages in frequency bandwidth than SDR IMPATT diode, and the upper limit of operating frequency is 50 GHz higher. Figure 9b shows the relationship between the negative resistance characteristics and the frequency of the two structures. As the frequency increases, the negative resistance gradually decreases. Under the same operating frequency, the negative resistance of the DDR IMPATT diode is slightly larger than that of the SDR IMPATT diode. 

**Figure 10:**
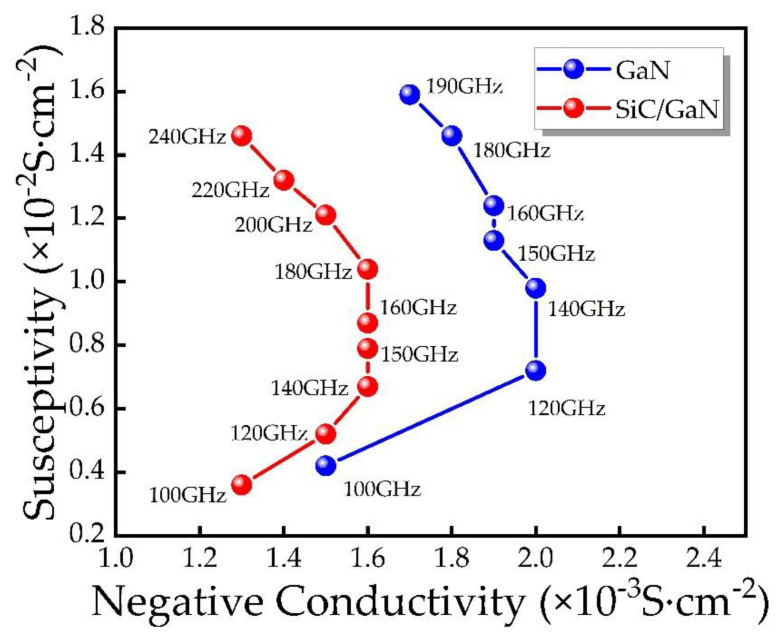
Admittance characteristics of IMPATT devices with different structures.

Figure 10 showcases the functional relationship between negative conductance and susceptance of IMPATT diodes with different structures. The admittance characteristics of the DDR IMPATT diode and the SDR IMPATT diode are similar. SDR IMPATT has the highest conductivity in the 120–140 GHz frequency band, DDR IMPATT has the highest conductivity in the 140–180 GHz frequency band, and the latter has a wider frequency band.

**Figure 11:**
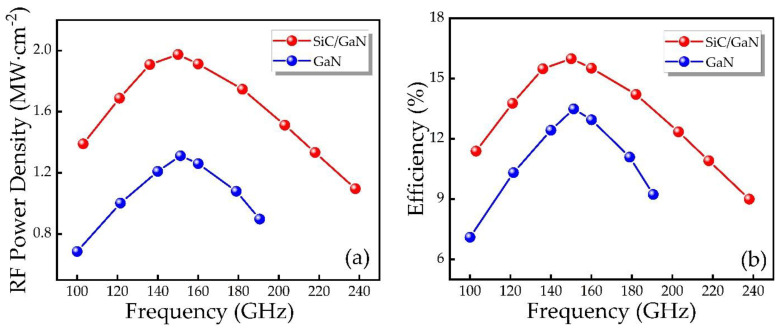
The relationship between (**a**) RF power, (**b**) efficiency and frequency of different IMPATT diodes.

Finally, we calculate the RF power and DC-RF conversion efficiency characteristics of the IMPATT devices of the two structures through the obtained parameters, as shown in Figure 11. Based on the results, the DDR IMPATT diode has higher RF power, larger bandwidth and better DC-RF conversion efficiency. Table 3 offers the specific values of the RF power, efficiency, RF current, RF voltage and phase delay of the two types of IM-PATT devices at the optimal frequency in the free-running oscillation circuit. The RF performance is mainly determined by the RF current, RF voltage and phase delay of the oscillating waveform. Due to the large DC breakdown voltage of DDR IMPATT diode, higher RF voltages can be obtained, thereby achieving greater RF power and higher efficiency. Through the matching design of the p-type drift region and the n-type drift region, the best frequency of the IMPATT diodes of the two structures is 150 GHz. The RF power and DC-RF conversion efficiency of DDR IMPATT diode at the optimal frequency of 150 GHz are 1.97 MW/cm^2^ and 15.99%, respectively, while SDR IMPATT is only 1.33 MW/cm^2^ and 13.49%. The RF output power of DDR IMPATT diode is significantly higher than that of SDR IMPATT diode, and the DC-RF conversion efficiency has also been improved to a certain extent.

With the help of the voltage-driven oscillator circuit in Figure 2b, we explores the output characteristics of the IMPATT devices of the two structures under different RF voltage modulation factors *m*. 

**Figure 12:**
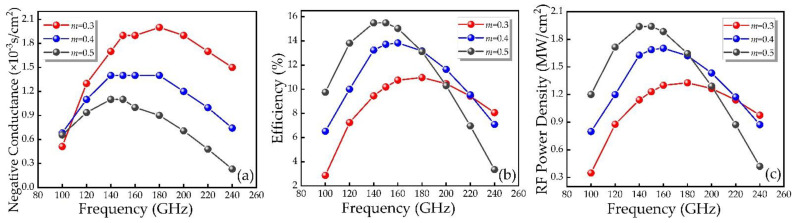
(**a**) Negative conductance, (**b**) efficiency and (**c**) RF power as a function of the frequency of the SiC/GaN DDR IMPATT under different voltage modulation factors *m*.

As can be seen from Figure 12, in the voltage-driven oscillation circuit, the voltage modulation factor *m* of the DDR IMPATT device is controlled by changing the applied voltage to obtain the conductance, RF power and efficiency as a function of frequency, respectively. In the 100–200 GHz frequency band, as the modulation factor *m* increases, the conductance gradually decreases, and the efficiency and RF power gradually increase. It implies that in the vicinity of the optimal frequency, the efficiency and RF power can be improved by increasing the RF voltage of the device.

**Figure 13:**
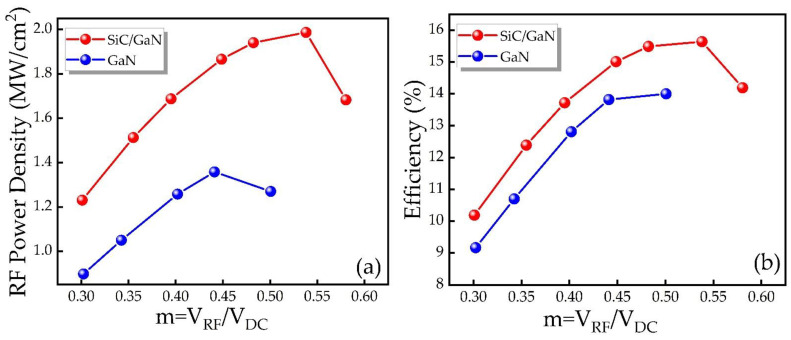
(**a**) RF power and (**b**) efficiency as a function of the voltage modulation factor *m* at 150 GHz.

Figure 13 provides the RF power and efficiency as a function of the voltage modulation factor *m* for SiC/GaN DDR and GaN SDR IMPATT at 150 GHz, respectively. They have the same change trend for the two structures. As the *m* rises, both the RF power and efficiency first increase, and then begin to decrease after reaching the saturation value. The RF performance of DDR and SDR IMPATT reach saturation values when *m* is 0.55 and 0.45, respectively. Obviously, it is important that the DDR IMPATT proposed in this design can withstand a larger *m* than the conventional SDR IMPATT, and has a significant improvement in RF power and efficiency.

**Figure 14:**
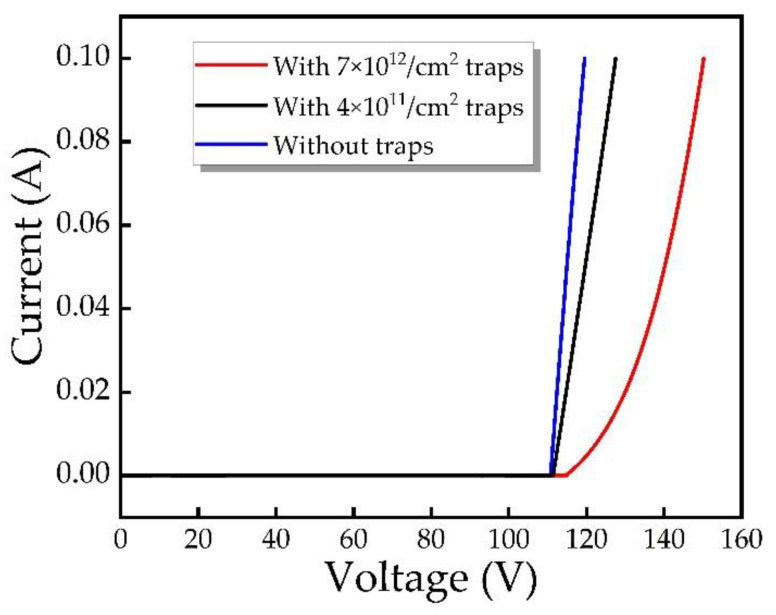
Reverse IV curve of DDR IMPATT with and without interface traps.

As a heterojunction device, the quality of the SiC/GaN interface does influence the device performance, for example, the interface mismatch, the interface state, and so on. Interface traps can affect the transport of electrons through the interface. Thus, the device performance degrades. However, due to the similar bandgap width of SiC and GaN and the very low lattice mismatch, which is only 3.4% [31], SiC is mature for GaN heteroepitaxy compared with Si, sapphire and other substrates [21,32,33]. According to previous studies [34], the main mechanism affecting the operating characteristics of SiC/GaN heterojunction diode is not the energy band or lattice mismatch, but the interface traps. We simulated the effect of interface fixed charge on the device performance, and found that compared to the large amount of charge produced by avalanche, the fixed interface charge cannot significantly affect the characteristics of the device. We also preliminarily simulated the device reverse IV curve with 7 × 10^12^ cm^−2^ [34,35], 4 × 10^11^ cm^−2^ and without interface traps. The results are shown in Figure 14, the breakdown voltages with 7 × 10^12^ cm^−2^, 4 × 10^11^ cm^−2^ and without interface traps are 119.5 V, 111.5 V and 110 V, respectively. At 100 kA/cm^2^ bias current, the device voltages with 7 × 10^12^ cm^−2^, 4 × 10^11^ cm^−2^ and without traps are 150 V, 127 V and 115 V, respectively. The DC voltage of the device with certain traps are higher, and this increase of DC operating voltage may be beneficial to the IMPATT, but more detailed investigation and discussion combined with other mechanisms are needed.

**Figure 15:**
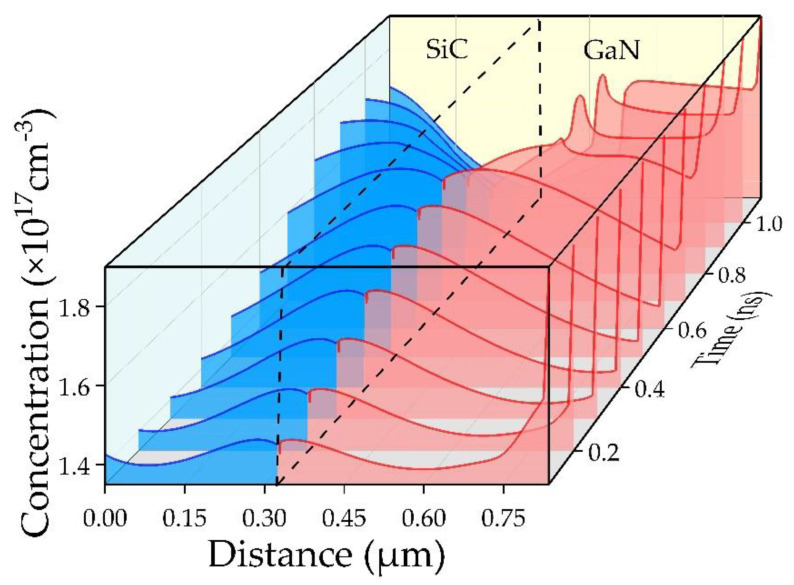
Concentration distribution of holes and electrons in the DDR IMPATT at a fixed time interval of one tenth period at 150 GHz.

Figure 15 shows the carrier concentration distribution curve of the p-SiC/n-GaN DDR IMPATT diode at a fixed time interval of one tenth of a period. The blue is the hole concentration curve in the p-SiC, and the red is the electron concentration curve in n-GaN. Different from the formation and transition of electron concentration packets that exist in SDR IMPATT diode, it can be clearly observed that the accumulation of hole charge packets and the transition process occur on the p-type SiC side. On the GaN side, due to the negative differential mobility characteristics of the material, a spike of electron packets appears, while on the SiC side, the morphology of the hole packets is consistent with the morphology in the conventional Si-based IMPATT.

## 4. Conclusions

This paper proposes a new type of p-SiC/n-GaN IMPATT diode based on a heterojunction, which adopts p-SiC instead of immature p-GaN to manufacture DDR IMPATT diodes and makes the design of DDR diodes more feasible. The research results prove that the new structure has obtained better RF output performance, manifested as a significant increase in RF power and an improvement in DC-RF conversion efficiency. According to the research results, the SiC/GaN DDR IMPATT diode proposed in this paper can obtain an RF power density of 1.97 MW/cm^2^ at a frequency of 150 GHz under a fixed DC bias current density, which is significantly higher than the SDR IMPATT diode under the same working conditions. In addition, the DC-RF conversion efficiency has also been enhanced to a certain extent. It can also be seen from the simulation results that the DDR IMPATT diode has a larger frequency bandwidth and better RF voltage modulation factor *m*. Consequently, it provides a new idea and reference for the design and manufacture of IMPATT devices on the basis of wide bandgap semiconductor materials.

## Figures and Tables

**Table 1:** Design parameters of different IMPATT diodes.

Structure	SiC/GaN DDR IMPATT						GaN SDR IMPATT			
Materials	SiC	SiC	SiC	GaN	GaN	GaN	GaN	GaN	GaN	GaN
Adulteration	p	p	p	n	n	n	p	n	n	n
Thickness (μm)	0.05	0.18	0.15	0.2	0.3	0.05	0.15	0.2	0.3	0.2
Concentration (cm^−3^)	1 × 10^19^	5 × 10^16^	1 × 10^18^	1 × 10^18^	5 × 10^16^	1 × 10^19^	1 × 10^19^	1 × 10^18^	5 × 10^16^	1 × 10^19^

**Table 2:** Parameters used in the simulation.

Materials	Parameter	Value	Parameter	Value
GaN	a_n_ (cm^−1^)	2.049 × 10^6^	a_p_ (cm^−1^)	2.04 × 10^6^
	b_n_ (V·cm^−1^)	1.27 × 10^7^	b_p_ (V·cm^−1^)	1.4 × 10^7^
	A	1 × 10^8^	B	1.9 × 10^7^
	V_sat_ (cm·s^−1^)	1.90 × 10^7^	E_C_ (kV·cm^−1^)	220.893
	δ	7.2044	γ	0.785
	a	6.1973	λ	2.5
SiC	a_n_ (cm^−1^)	1.66 × 10^6^	a_p_ (cm^−1^)	5.18 × 10^6^
	b_n_ (V·cm^−1^)	1.28 × 10^7^	b_p_ (V·cm^−1^)	1.4 × 10^7^
	V_satn_ (cm·s^−1^)	2 × 10^7^	V_satp_ (cm·s^−1^)	1 × 10^6^

**Table 3:** Comparison of RF output performance of different IMPATT diodes.

Structure	Optimum Frequency (GHz)	Efficiency (%)	Power (MW/cm^2^)	V_RF_ (V)	I_RF_ (A)	Phase Delay (°)
SDR IMPATT	150	13.49	1.33	37.02	0.43	99.60
DDR IMPATT	150	15.99	1.97	50.4	0.40	101.06
